# CHOIRBM: An R package for exploratory data analysis and interactive visualization of pain patient body map data

**DOI:** 10.1371/journal.pcbi.1010496

**Published:** 2022-10-27

**Authors:** Eric Cramer, Maisa Ziadni, Kristen Hymel Scherrer, Sean Mackey, Ming-Chih Kao

**Affiliations:** 1 Division of Pain Medicine, Stanford University School of Medicine, Palo Alto, California, United States of America; 2 Department of Cell Biology and Physiology, University of North Carolina at Chapel Hill School of Medicine, Chapel Hill, North Carolina, United States of America; bioinformatics, GERMANY

## Abstract

Body maps are commonly used to capture the location of a patient’s pain and thus reflect the extent of pain throughout the body. With increasing electronic capture body map information, there is an emerging need for clinic- and research-ready tools capable of visualizing this data on individual and mass scales. Here we propose CHOIRBM, an extensible and modular R package and companion web application built on the grammar of graphics system. CHOIRBM provides functions that simplify the process of analyzing and plotting patient body map data integrated from the CHOIR Body Map (CBM) at both individual patient and large-dataset levels. CHOIRBM is built on the popular R graphics package, ggplot2, which facilitates further development and addition of functionality by the open-source development community as future requirements arise. The CHOIRBM package is distributed under the terms of the MIT license and is available on CRAN. The development version of the package with the latest functions may be installed from GitHub. Example analysis using CHOIRBM demonstrates the functionality of the modular R package and highlights both the clinical and research utility of efficiently producing CBM visualizations.

## Author summary

The number of patients with chronic pain conditions has steadily and dramatically increased over time, leading to immense individual and societal burden. To better study and improve treatments for these conditions, it is important to develop methods for characterizing the patients’ pain. Central to this effort is describing the location and distribution of pain throughout each patient’s body. Body maps are visual methods that efficiently and effectively facilitate capturing the location and extent of a patient’s pain and can be readily integrated with electronic data capture systems. As electronic health records have become the cornerstone of patient care, there is an emerging need for clinic- and research-ready tools to visualize body-map data on individual and mass scales. To address this need, Stanford researchers developed and validated the CHOIR Body Map for capturing the locations and distribution of a given patient’s pain, and we developed the CHOIRBM R package for analyzing the data. The CHOIRBM software provides functions for analyzing or visualizing individual body maps and large-scale data sets for comparisons across groups such as demographics or pain conditions. In addition, we built CHOIRBM with the popular R graphics package ggplot2 to facilitate further development or customization as future needs arise.

This is a *PLOS Computational Biology* Software paper.

## Introduction

There is a critical need to better characterize and manage pain in light of chronic pain’s immense individual and societal burden [1–4]. Central to pain characterization is the location and distribution of pain throughout the body [1,2]. Several dedicated efforts to develop body maps [1–5] face limitations, including low resolution, condition-specific features, anatomical demarcations not corresponding to clinical pain conditions, or paper and pencil requirements. To address the need for a standardized, digital, general-purpose body map to collect self-reported pain location data efficiently, Stanford researchers developed and validated the CHOIR body map (CBM) [6], as part of CHOIR, an open-source electronic learning healthcare system [7,8].

The CHOIR platform uses item-response theory-based measures, including the National Institute of Health’s (NIH) Patient-Reported Outcomes Measurement Information System (PROMIS), which was designed and validated for precise and efficient measurement of health-related symptoms in patients with a wide variety of chronic conditions [9]. Recently, a formal initial validation demonstrated that the CBM possessed validity, reliability, and utility as an instrument to efficiently collect data on self-reported pain location and distribution and is thus a cost-effective diagnostic and prognostic tool [6]. Furthermore, as the CBM is multifunctional, it may be used to address conditions relating to nociceptive pain (caused by inflammation), neuropathic pain (caused by nerve damage), and nociplastic pain (diffuse pain not associated with inflamed tissue or nerve damage) [6,10,11].

Together, the CHOIR platform and integrated body map provide a multi-purpose, digital tool to facilitate comprehensive, multidimensional pain assessment, characterization, and visualization to inform large-scale pain characterization research and clinical efforts.

Currently, over 100,000 CBM assessments have been collected and analyzed [7,8,12,13,13–30] through CHOIR, across institutions and clinical sites worldwide. In addition to the multi-site CHOIR electronic data capture ecosystem, the CBM has also been integrated into research workflows such as Research Electronic Data Capture (REDCap), a cloud-based, secure software [30] application for clinical research. The extensive, multi-site use of the CBM for research and medical purposes since 2013 has led to the creation of large data sets. However, a tool is not readily available to generate, analyze, and visualize body map- and integrated- data. This makes finding data-driven insights cumbersome and leads to non-standard methods of analysis. Thus, there is a demonstrable need for an informatics tool to analyze body map data that will aid researchers and clinicians seeking to understand the anatomical location, distribution, and comorbidities of their patients’ pain.

This manuscript introduces CHOIRBM, an R package that provides a collection of functions for data formatting, processing, and visualizing anatomical pain data using the CBM. Novel aspects of the package include: a suite of plotting methods to enable efficient and flexible visualization of complex and large body map data sets through an Application Programming Interface (API) and several functions for statistical comparisons and tests. In addition, it is the first tool to generate a colored body map, provide tools for comparing body maps across groups, and methods for analyzing the effect of continuous variables (such as NIH PROMIS measures) on body map endorsement. The intended users of this R package are researchers, statisticians, and clinicians interested in analyzing an individual patient or large body map data provided for pain characterization. In this paper, we demonstrate the use of this novel R package using data from the original CBM validation study collected through REDCap [6]. These analyses demonstrate the core functionality of the package and highlight both the clinical and research utility of efficiently producing CBM visualizations.

## Methods

### CHOIR body map data capture

**Fig 1:**
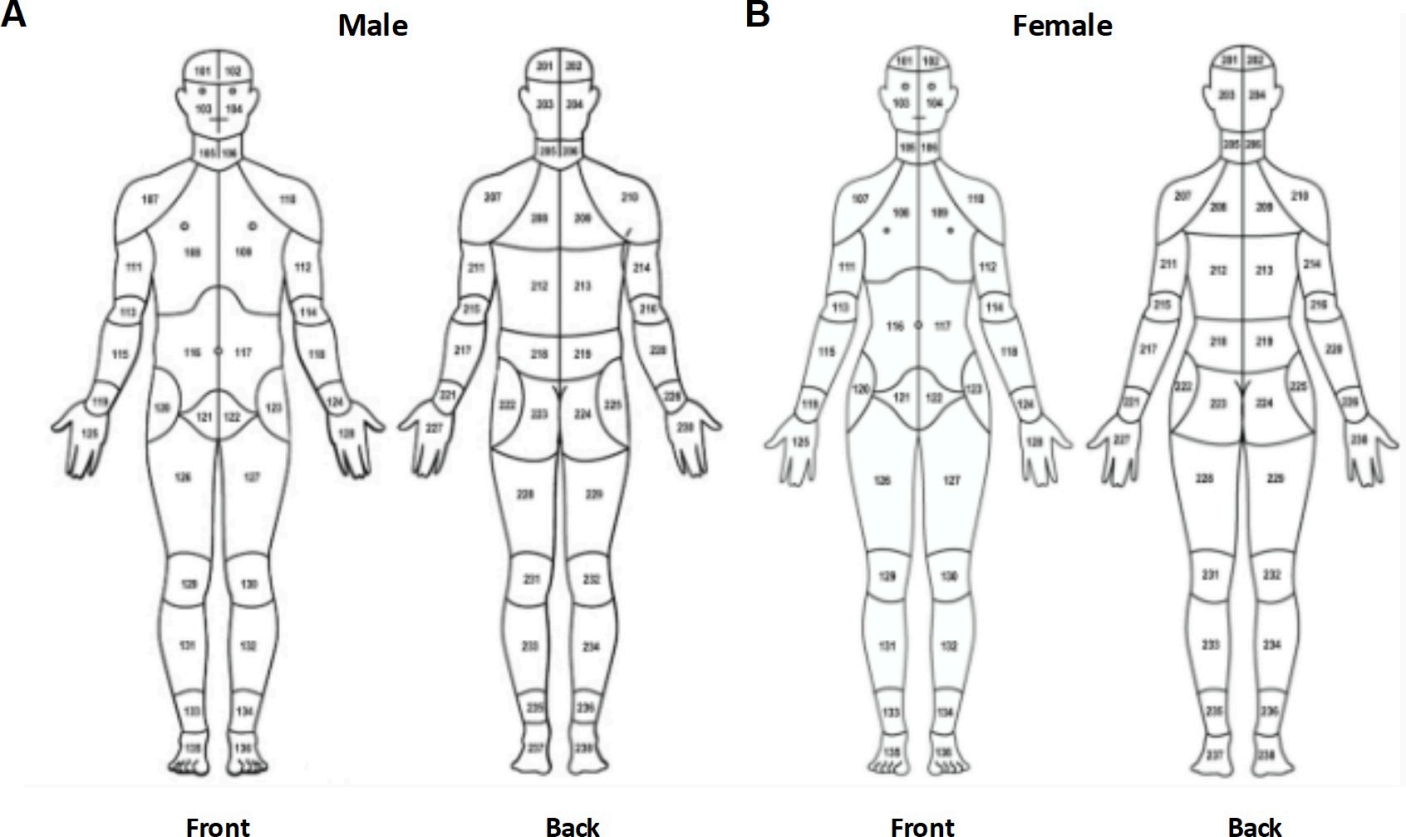
The (A) male and (B) female CBM with each body map area labeled with its three-digit identification code.

The CBM is an electronic, visual representation of the human body that enables participants to indicate the location(s) of their pain (**Fig 1**). Participants use a computer mouse or touchscreen device to select each body area in which they experience pain. The CBM has two body silhouettes of identical segmentation to reflect the female and male anatomy. Each silhouette has 36 anterior and 38 posterior symmetrical body segments that best align with typical distributions of common chronic pain conditions on the body surface and joints. Each of the 74 anatomical locations for pain endorsement is identified by a three-digit ID code for efficient data capture and analysis. Codes that begin with a 1 correspond to locations on the front of the body while codes that begin with a 2 correspond to locations on the back of the body. Note to users, the three-digit identification codes differ between the male and female silhouettes, however, the CHOIRBM R package has functions to match them (functions convert_bodymap() and convert_bodymaps()).

### Design and implementation

The CHOIRBM package was designed to be open-source and built on top of the application programming interface (API) of the popular R data visualization package, ggplot2. Therefore, CHOIRBM is implemented in an object-oriented manner, with a series of functions that operate on base R objects such as data.frames and lists to produce ggplot2 objects. This approach makes the CHOIRBM API intuitive to users familiar with the R programming language and facilitates efficient and straightforward plot customization.

The standard analysis workflow is to import the dataset as an R data.frame, use CHOIRBM helper functions to reformat the data to match relevant values to specific locations on the CBM (if necessary), use built-in analytic tools to compare and derive clinical insights, and use the plotting functions to generate publication-ready figures.

We implemented CHOIRBM to include basic analytic functions: to compare CBMs across groups (e.g., male versus female, two groups with different pain conditions, or two time points), to investigate the impact of continuous variables on body map endorsement (e.g., age, NRS pain scores, or PROMIS measures), and to create plots to derive insights from the dataset, as demonstrated herein visually. Documentation of all functions organized by capability and additional details and example workflows can be found in the package vignettes online (https://www.github.com/emcramer/CHOIRBM).

### Data format and processing

CHOIRBM can process CBM data from two different data sources: the CHOIR database which uses SQL tables or REDCap. In each case, data is imported into the R programming language and stored in computer memory as an R data.frame (analogous to an Excel spreadsheet).

CHOIRBM does not introduce any package-specific data structures or objects. Thus, the primary data class in the CHOIRBM package is a data.frame with a minimum of three columns: [1] a column indicating the three-digit identification number of a CBM location, [2] a grouping column indicating if the location is on the front or back of the CBM, and [3] a column containing the values to use for coloring and filling the CBM locations in the plot. This data.frame-based approach simplifies the process of visualizing information by directly loading data from any spreadsheet, delimited file, R data file, or SQL query, and ensures flexibility by allowing users to easily switch values for plotting. For example, the percent endorsement, raw count, or any other measure or score. Therefore, plotting functions in the CHOIRBM package are written to operate on data.frame objects and work with R tidyverse pipes.

### Working with data extracted from a CHOIR database

**Fig 2:**
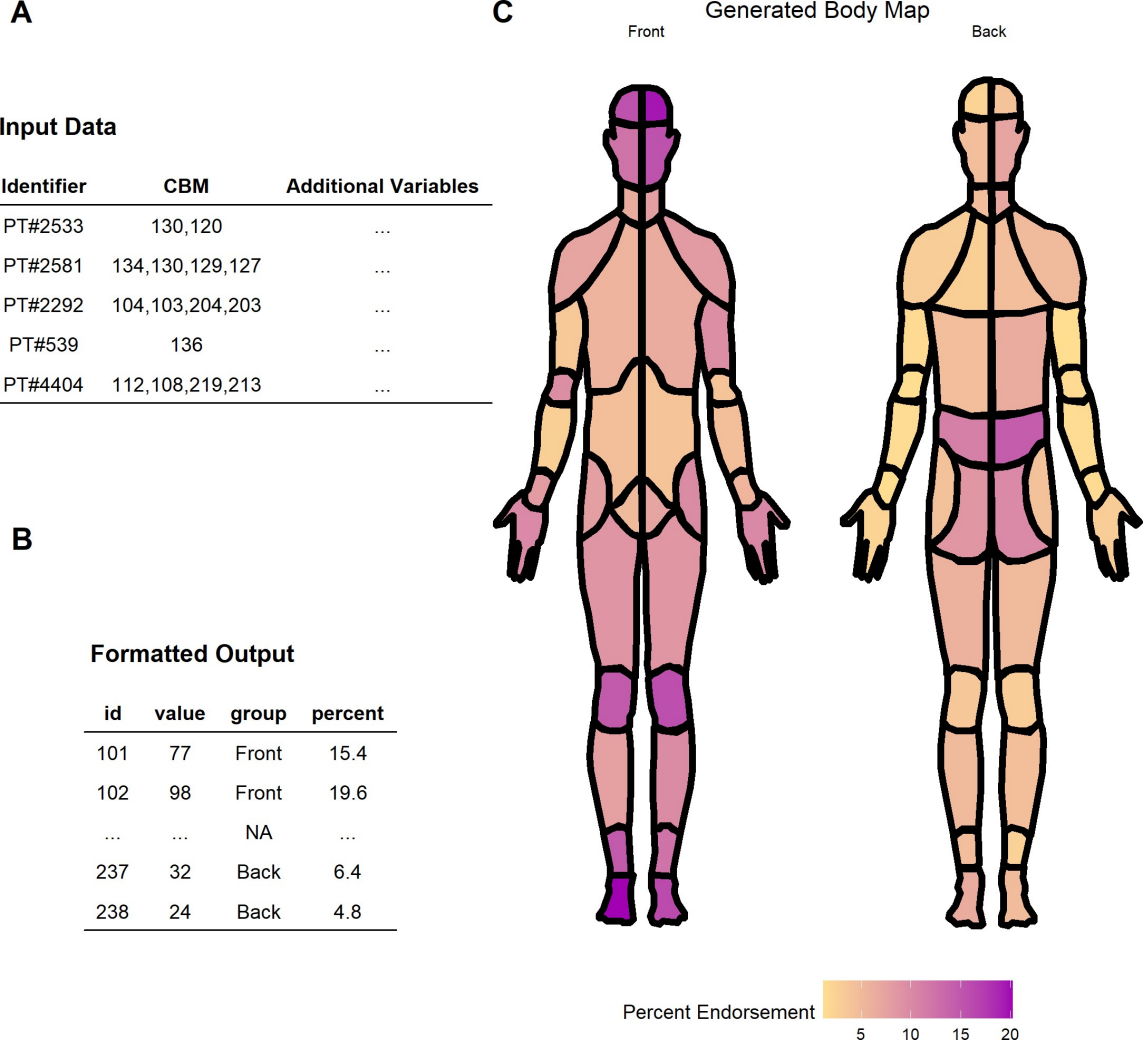
(A) Example of the format for an input data.frame for the CHOIRBM package. (B) Example of an output data.frame ready for plotting. Note, only the first two rows and last two rows are shown. (C) Example data from (A) and (B) plotted in a CBM.

The CHOIR interface for the CBM consists of a clickable CBM image. Each anatomical location that the patient selects is recorded by CHOIR as a series of thee-digit codes in a delimited string. CBM data extracted from CHOIR databases is obtained as a series of pain location identifiers in a comma-separated string; with one string for each patient in a dataset. The data is exported from CHOIR with an SQL query and is automatically in R tidy format, with each row in the table representing a patient or participant and each column representing a variable; including each patient’s CBM endorsement (**Fig 2A**).

The data can be transformed from the raw delimited body map strings using the string_to_map() function. string_to_map() will create a single body map data.frame from a patient’s string indicating binary endorsement of different body map segments. These individual body maps can be aggregated with the aggregate_maps() function, which accepts a list of CBMs and sums the endorsement of each anatomical location across all possible locations to produce a single data.frame with the raw count ready to plot as shown in **Fig 2B**, and the resulting visualization of CBM data in **Fig 2C**.

### Working with data extracted from a REDCap project

**Fig 3:**
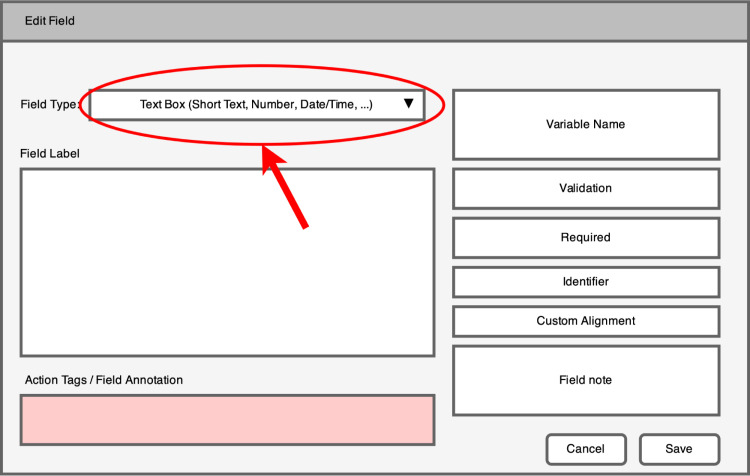
Example of the CBM instrument format required in REDCap for streamlined use with the CHOIRBM package. Selecting a single text box to collect a patient’s body map data allows the CHOIRBM string_to_map() function to automatically generate plot-ready R data.frames.

The REDCap interface for the CBM also consists of a clickable CBM image and each anatomical location that the patient selects on the clickable image-map is recorded by the REDCap system. Importantly, however, the data format is determined by how a researcher programs the CBM instrument into their REDCap project. A patient’s CBM may be recorded in REDCap as either a series of thee-digit codes in a delimited string (similar to the method of export for CHOIR databases), or a collection of check boxes which results in 74 one-hot encoded variables in the exported dataset. While REDCap allows the user to choose which method to use, CHOIRBM will *only* accept data from REDCap that has been formatted in a delimited string, and researchers *must* program their CBM instrument to use a text-box field as outlined in **Fig 3** (which produces a delimited string). By following this convention, data files exported from REDCap via manual download or its API will be formatted appropriately (**Fig 2A**) for immediate use with the CHOIRBM string_to_map() function, thereby reducing the need for data quality control.

The data will be exported in R tidy format, with each row representing a patient and each column containing a variable (with one column for CBM endorsement). The string_to_map() function will create a single body map data.frame from a patient’s string indicating binary endorsement of different body map segments. These individual body maps can be aggregated with the aggregate_maps() function, which accepts a list of CBMs and sums the endorsement of each anatomical location across all possible locations to produce a single data.frame with the raw count ready to plot as shown in **Fig 2B**, and the resulting visualization of CBM data in **Fig 2C**.

### Analysis

There are multiple ways to analyze CBM data depending on the variables of interest or the research question. The CHOIRBM package includes the following quantitative methods for analyzing body map endorsement information: 1) inter-group comparisons with a categorical variable such as gender, pain condition, or time point, 2) measuring the association of a continuous variable such as pain intensity scores or an NIH PROMIS measure with body map location endorsement, and 3) identifying co-occurrence patterns in body map location endorsement.

### Inter-group comparisons with a categorical variable

For comparing body map endorsement between groups using a variable with two categories such as gender or time point, CHOIRBM includes the comp_choirbm_ztest() function. This function takes as input two R data.frames, one for each group. The data.frames are in R tidy format, with each row in the table representing a patient or participant, and each column representing a variable with one of those columns containing that individual’s CBM endorsement as a delimited string. The program then runs a series of z-tests to test whether there are statistically significant differences in endorsement of each location on the body map between groups [30]. To account for multiple hypothesis testing, comp_choirbm_ztest() automatically adjusts the p-values using the Bonferroni correction procedure, or users have the option to supply their own correction method. Users may also choose between left, right, and two-tailed z-tests to investigate the directionality of each relationship. The function returns a data.frame with one row for each anatomical location on the CBM, and columns for the z-test’s z-score and p-value.

### Measuring the impact of a continuous variable on CBM location endorsement

For investigating the effect of a continuous variable such as pain intensity score or an NIH PROMIS measure on CBM segment endorsement, CHOIRBM includes the comp_choirbm_glm() function. comp_choirbm_glm() accepts a data.frame with at least one column for the patients’ CBM endorsement in a delimited string, and another column with the variable of interest. The function returns a data.frame object where each row is the result of a logistic regression examining the relationship between the continuous variable and patient endorsement [30]. Similar to comp_choirbm_ztest(), the p-values are adjusted with the Bonferroni correction by default to account for multiple hypothesis testing but the correction method may be changed at the user’s discretion.

### Investigating co-occurrence of CBM location endorsement

CBM co-occurrence is defined as the number of times two anatomical locations on the CBM are endorsed together by patients in a data set. For example, given two patients where one endorses the locations numbered "101, 102, 103, 104, 201, 202" and the other indicates "101, 102, 201, 202," the location coded "101" co-occurs with "103" and "104" once, but with "102", "201", and "202" twice. Co-occurrence plays a role in chronic overlapping pain conditions (COPCs) and may be used to determine whether pain locations are more commonly endorsed together due to a particular etiology or pathology [31].

CHOIRBM supports co-occurrence analysis with the comp_cooccurrence() function. comp_cooccurrence() accepts a data.frame in R tidy format where one of the columns contains the patients’ CBM endorsements as delimited strings. It then calculates the number of times any two CBM segments are observed together in each body map across the entire data set. The function returns a data.frame object where each row is a combination of locations and a column that contains the number of times each combination of CBM locations occurred together (co-occurrence).

### Data visualization

CHOIRBM includes four main visualization functions: plotting the front and back of the male or female CBM, the distribution of the number of CBM location endorsements, as well as a heatmap of CBM location co-occurrence. The plot_male_choirbm() and plot_female_choirbm() functions accept data.frames with one row for each location of the CBM, and a minimum of three columns: [1] a column indicating the three-digit identification number of the CBM location, [2] a grouping column indicating if the location is on the front or back of the CBM, and [3] a column containing the values to use for coloring and filling the CBM locations in the plot. An example of the input data.frame is shown in **Fig 2B**.

The plot_nareas_histogram() function in CHOIRBM enables users to view the distribution of the number of locations each patient endorses. It accepts a vector of body maps in the form of delimited strings and produces a histogram. Users can control the number of bins or the width of the bins in the histogram using standard ggplot2 arguments.

In addition, the co-occurrence of pain locations on the CBM can be visualized with the plot_cooccurrence() function, which is designed to accept the output of comp_cooccurrence(). This generates a heatmap visual of which CBM locations most frequently occur together in the data set.

Since CHOIRBM was developed with the ggplot2 package, the resulting plot objects operate within the grammar of a graphics system [30]. Therefore, the aesthetic of the plots can be easily customized to suit the needs of each user. The visualizations can be enhanced with interactivity by using the R plotly package to generate web-friendly interactive graphics.

## Results

We demonstrate the primary data processing, analysis, and visualization functionality possible with CHOIRBM using the dataset obtained during the validation of the CBM instrument (and for which a permuted and de-identified version is built-into the R package). Detailed information about the dataset, including the study design, acquisition process, and population characteristics are described elsewhere [6]. Data were imported into R version 4.0.3 and the development version of the CHOIRBM package available on GitHub was loaded into the R namespace. Below we provide examples of the CHOIRBM’s analytical functions and data visualizations.

### CBM endorsement distribution

**Fig 4:**
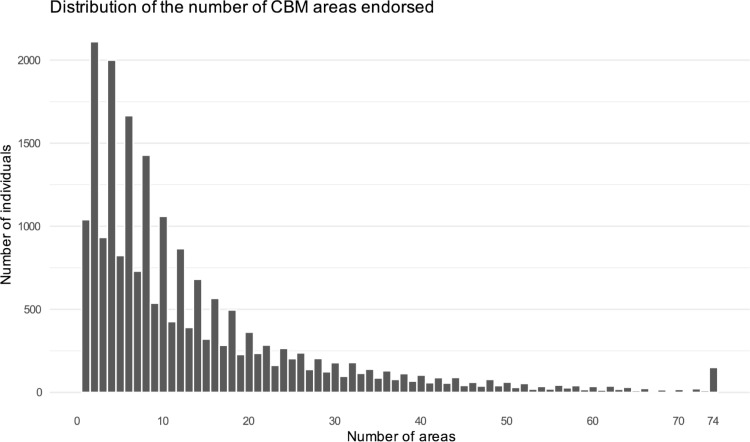
The distribution of the number of areas on the CBM (each bar represents one value) that each patient endorses can be visualized with the plot_nareas_histogram() function.

To illustrate a histogram data visualization from an extracted dataset, the distribution of the number of body map locations endorsed by patients was plotted with the plot_nareas_histogram() function, and is shown in **Fig 4**. We observed a right-skewed distribution with most patients endorsing between one and ten locations on the CBM, which suggests our dataset may contain patients with predominantly localized pain.

### Inter-group comparisons with gender

**Fig 5:**
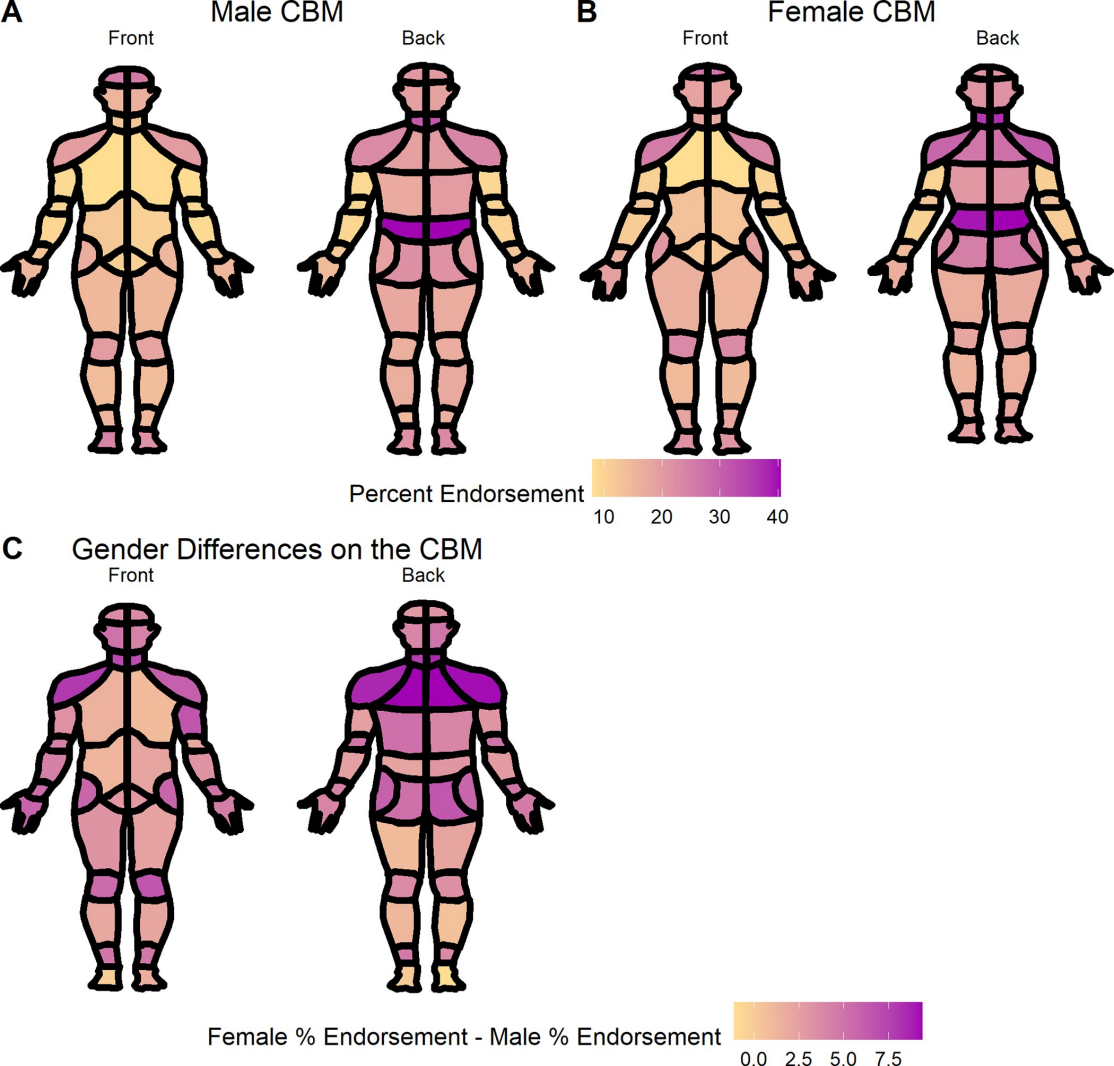
The (A) male and (B) female CBM with the percentage of patients who endorsed each body location. (C) The difference between the percent female endorsement of each CBM location and the percent male endorsement (subtract male endorsement from female endorsement).

To compare the proportion of men endorsing each location on the CBM to the proportion of women, the data was split into two data.frames, one for each gender. The comp_choirbm_ztest() function was used to determine whether the proportion of men endorsing a given CBM location was less than the proportion of women endorsing the same location. This comparison, shown in **Table 1**, indicates that greater proportions of women endorse all areas of the body map except for the top of the head, chest, calves, and feet (location codes 101, 102, 108, 109, 135, 136, 233, 234, 237, and 238 with p-values < 0.05). The plot_male_choirbm() and plot_female_choirbm() functions were then used to visualize the percentage endorsement of each CBM location by gender, and the differences between gender (**Fig 5).** These results support the clinical observation of chronic lower back and spinal pain among men and women [32,33], and indicate that women may endorse greater shoulder and hip pain when compared to men [34,35].

**Table 1:** The results of a left-tailed z-test to determine whether the proportion of men endorsing each body map area was less than the proportion of women endorsing the same area. The p-values were adjusted for multiple hypothesis testing with the Bonferroni correction (the default for the package function comp_choirbm_ztest()). Location codes that start with a “1” indicate the front of the body and codes that begin with a “2” indicate the back of the body.

CBM Area ID Number	Anatomical Description	Z Score	p-value
101	Top of the Head	-3.1994	0.050955
102	Top of the Head	-3.18658	0.053268
103	Face	-5.21344	6.86E-06
104	Face	-4.29583	6.44E-04
105	Neck	-7.46699	3.04E-12
106	Neck	-7.41055	4.65E-12
107	Right Shoulder	-7.26133	1.42E-11
108	Chest	-2.0909	1
109	Chest	-1.32834	1
110	Left Shoulder	-5.69128	4.67E-07
111	Right Upper Arm	-4.23663	8.40E-04
112	Left Upper Arm	-7.8358	1.72E-13
113	Right Elbow	-5.08317	1.37E-05
114	Left Elbow	-2.67162	0.279299
115	Right Forearm	-5.18998	7.78E-06
116	Abdomen	-1.49866	1
117	Abdomen	-2.77076	0.206926
118	Left Forearm	-4.11997	0.001402
119	Right Wrist	-5.73973	3.51E-07
120	Right Hip	-5.60095	7.89E-07
121	Pelvis	-3.49073	0.017823
122	Pelvis	-3.49889	0.017286
123	Left Hip	-5.82834	2.07E-07
124	Left Wrist	-5.49969	1.41E-06
125	Right Hand	-5.94405	1.03E-07
126	Right Upper Leg	-3.40929	0.024099
127	Left Upper Leg	-2.54064	0.409409
128	Left Hand	-4.57461	1.77E-04
129	Right Knee	-5.14975	9.65E-06
130	Left Knee	-6.23186	1.71E-08
131	Right Lower Leg	-2.25302	0.897533
132	Left Lower Leg	-2.41738	0.578407
133	Right Ankle	-4.92646	3.10E-05
134	Left Ankle	-4.79111	6.14E-05
135	Right Foot	0.193361	1
136	Left Foot	-1.47442	1
201	Top of the Head	-2.74528	0.223698
202	Top of the Head	-2.94028	0.121328
203	Back of the Head	-3.80654	0.005214
204	Back of the Head	-4.7544	7.36E-05
205	Neck	-6.36756	7.11E-09
206	Neck	-6.95837	1.27E-10
207	Left Shoulder	-7.51429	2.12E-12
208	Upper Back	-8.52212	5.80E-16
209	Upper Back	-8.43234	1.25E-15
210	Right Shoulder	-7.98504	5.20E-14
211	Left Upper Arm	-3.35243	0.029638
212	Mid-Back	-5.06294	1.53E-05
213	Mid-Back	-3.84617	0.004439
214	Right Upper Arm	-3.87438	0.003955
215	Left Elbow	-5.81884	2.19E-07
216	Right Elbow	-5.8328	2.02E-07
217	Left Forearm	-3.18185	0.054145
218	Lower Back	-1.77968	1
219	Lower Back	-2.44107	0.541818
220	Right Forearm	-3.38627	0.026215
221	Left Wrist	-4.62307	1.40E-04
222	Left Hip	-5.67198	5.22E-07
223	Buttocks	-4.75292	7.42E-05
224	Buttocks	-5.90682	1.29E-07
225	Right Hip	-5.34728	3.30E-06
226	Right Wrist	-5.28094	4.76E-06
227	Left Hand	-4.36024	4.81E-04
228	Left Upper Leg	-0.88767	1
229	Right Upper Leg	-2.29902	0.795645
230	Right Hand	-4.68982	1.01E-04
231	Left Knee	-3.83037	0.004734
232	Right Knee	-3.44382	0.021222
233	Left Lower Leg	-1.21058	1
234	Right Lower Leg	-0.60771	1
235	Left Ankle	-4.79662	5.97E-05
236	Right Ankle	-3.91084	0.003403
237	Left Foot	0.047916	1
238	Right Foot	1.122213	1

The impact of pain intensity and emotional support on CBM location endorsement was investigated with the comp_choirbm_glm() function for each variable. The function assessed whether a patient’s average reported pain intensity (NRS scale from 1–10) or PROMIS Emotional Support (standardized t-score; *M* = 50, *SD* = 10) were predictive of CBM area endorsement. The results shown in **Table 2** indicate that higher pain intensity scores predict increased CBM location endorsement for all CBM locations except for the top of the head and front of the face (location codes 101, 102, 103, and 104 with p-values < 0.001). The CBM locations 101, 102, 103, and 104 showed negative correlations with, and were not significantly predicted by, the average pain intensity score.

**Table 2:** The results of logistic regression models for each CBM location to quantify the relationship between average pain intensity score and endorsement of each location. Location codes that start with a “1” indicate the front of the body and codes that begin with a “2” indicate the back of the body.

CBM Area ID Number	Anatomical Description	Coefficient Estimate	p-value
101	Top of the Head	-0.01107	1
102	Top of the Head	-0.00797	1
103	Face	-0.02326	0.443504
104	Face	-0.01001	1
105	Neck	0.126388	9.18E-45
106	Neck	0.129446	5.22E-45
107	Right Shoulder	0.160981	9.01E-91
108	Chest	0.139626	3.16E-27
109	Chest	0.149383	1.22E-31
110	Left Shoulder	0.171763	2.76E-98
111	Right Upper Arm	0.220339	2.37E-81
112	Left Upper Arm	0.114717	1.80E-28
113	Right Elbow	0.132365	1.76E-36
114	Left Elbow	0.220882	1.78E-76
115	Right Forearm	0.215787	3.04E-88
116	Abdomen	0.215456	5.91E-86
117	Abdomen	0.194597	6.09E-69
118	Left Forearm	0.194293	5.34E-64
119	Right Wrist	0.159801	2.91E-64
120	Right Hip	0.154408	1.96E-72
121	Pelvis	0.105326	1.37E-22
122	Pelvis	0.107342	1.18E-22
123	Left Hip	0.160324	2.95E-73
124	Left Wrist	0.166136	5.29E-65
125	Right Hand	0.150919	1.17E-65
126	Right Upper Leg	0.195028	3.18E-93
127	Left Upper Leg	0.219427	5.36E-111
128	Left Hand	0.154231	8.69E-64
129	Right Knee	0.161524	5.31E-86
130	Left Knee	0.167056	7.83E-91
131	Right Lower Leg	0.200655	1.24E-89
132	Left Lower Leg	0.208188	6.53E-95
133	Right Ankle	0.161473	4.44E-72
134	Left Ankle	0.170975	1.07E-77
135	Right Foot	0.134712	6.03E-59
136	Left Foot	0.147151	1.33E-67
201	Top of the Head	0.037267	2.91E-04
202	Top of the Head	0.036598	4.29E-04
203	Back of the Head	0.040551	4.28E-05
204	Back of the Head	0.033902	0.00176
205	Neck	0.088697	1.34E-35
206	Neck	0.079271	6.36E-29
207	Left Shoulder	0.141056	1.63E-77
208	Upper Back	0.117205	1.08E-50
209	Upper Back	0.112247	6.71E-48
210	Right Shoulder	0.131635	5.84E-70
211	Left Upper Arm	0.220112	2.05E-76
212	Mid-Back	0.174791	2.24E-91
213	Mid-Back	0.156337	1.87E-77
214	Right Upper Arm	0.218158	1.31E-80
215	Left Elbow	0.205592	2.23E-82
216	Right Elbow	0.203575	1.97E-84
217	Left Forearm	0.196678	2.78E-64
218	Lower Back	0.166258	2.08E-123
219	Lower Back	0.169834	1.81E-129
220	Right Forearm	0.190439	4.10E-65
221	Left Wrist	0.182184	4.91E-74
222	Left Hip	0.155662	3.04E-79
223	Buttocks	0.140022	4.07E-69
224	Buttocks	0.13384	5.93E-65
225	Right Hip	0.147416	6.65E-74
226	Right Wrist	0.171425	1.09E-70
227	Left Hand	0.155909	3.87E-62
228	Left Upper Leg	0.232292	4.36E-131
229	Right Upper Leg	0.203576	2.63E-106
230	Right Hand	0.152979	3.08E-64
231	Left Knee	0.217918	7.22E-125
232	Right Knee	0.222662	3.21E-131
233	Left Lower Leg	0.205996	1.84E-102
234	Right Lower Leg	0.205241	4.32E-103
235	Left Ankle	0.195287	3.11E-98
236	Right Ankle	0.184543	2.62E-92
237	Left Foot	0.170635	6.85E-85
238	Right Foot	0.152187	3.54E-70

**Fig 6:**
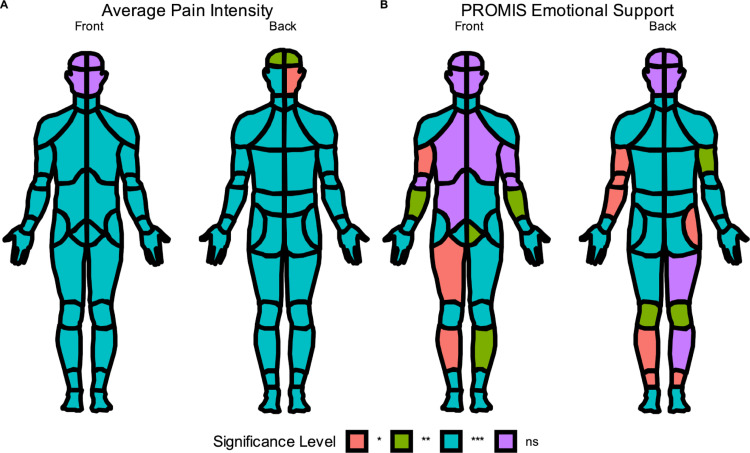
(A) Logistic regression results indicating which locations on the CBM were significantly predicted by the average pain intensity score. Significance levels were stratified and broken up into * p < 0.05, ** p < 0.01, *** p < 0.001, and ns p > 0.05. (B) Logistic regression results indicating which locations on the CBM were significantly predicted by the PROMIS Emotional Support score.

The PROMIS Emotional Support T Score predicted more specific locations of the CBM. As shown in **Table 3**, there is no relationship between Emotional Support and endorsement of the head areas, but significant relationships were found for the upper and lower back (p-values < 0.001), with other CBM areas showing statistically significant associations as well (p-values < 0.05). For the purposes of visualization, the resulting p-values for each measure were stratified by magnitude (< 0.05, < 0.001, < 0.0001). The plot_male_choirbm() function was then used to illustrate which CBM areas were statistically significantly predicted by average pain intensity or PROMIS Emotional Support (**Fig 6A and 6B,** respectively).

**Table 3:** The results of logistic regression models for each CBM location to quantify the relationship between PROMIS Emotional Support scores and endorsement of each location.

CBM Area ID Number	Anatomical Description	Coefficient Estimate	p-value
101	Top of the Head	4.47E-04	1
102	Top of the Head	-0.00105	1
103	Face	0.00143	1
104	Face	-0.00319	1
105	Neck	-0.0137	2.12E-09
106	Neck	-0.0152	3.34E-11
107	Right Shoulder	-0.01912	2.28E-23
108	Chest	-0.00509	1
109	Chest	-0.00638	1
110	Left Shoulder	-0.01493	1.07E-13
111	Right Upper Arm	-0.0102	0.007553
112	Left Upper Arm	5.05E-04	1
113	Right Elbow	-0.00226	1
114	Left Elbow	-0.00704	0.67916
115	Right Forearm	-0.01193	1.02E-04
116	Abdomen	-0.008	0.090067
117	Abdomen	-0.01395	3.11E-06
118	Left Forearm	-0.01156	7.99E-04
119	Right Wrist	-0.01773	2.44E-14
120	Right Hip	-0.01013	1.90E-05
121	Pelvis	-0.01186	9.39E-05
122	Pelvis	-0.01086	9.17E-04
123	Left Hip	-0.01118	2.52E-06
124	Left Wrist	-0.01529	5.64E-10
125	Right Hand	-0.02274	5.00E-27
126	Right Upper Leg	-0.00885	0.003277
127	Left Upper Leg	-0.0115	1.57E-05
128	Left Hand	-0.02004	1.21E-19
129	Right Knee	-0.01813	6.91E-20
130	Left Knee	-0.01784	5.06E-19
131	Right Lower Leg	-0.00899	0.005096
132	Left Lower Leg	-0.01087	1.33E-04
133	Right Ankle	-0.01123	3.45E-06
134	Left Ankle	-0.01192	1.08E-06
135	Right Foot	-0.01687	8.72E-17
136	Left Foot	-0.01542	2.21E-13
201	Top of the Head	-6.46E-04	1
202	Top of the Head	0.002589	1
203	Back of the Head	-0.00359	1
204	Back of the Head	-0.00155	1
205	Neck	-0.01352	1.95E-14
206	Neck	-0.01324	5.96E-14
207	Left Shoulder	-0.01358	6.44E-13
208	Upper Back	-0.01454	8.50E-14
209	Upper Back	-0.01458	3.47E-14
210	Right Shoulder	-0.01729	1.29E-21
211	Left Upper Arm	-0.00918	0.044197
212	Mid-Back	-0.01019	1.86E-05
213	Mid-Back	-0.01033	6.99E-06
214	Right Upper Arm	-0.01254	1.05E-04
215	Left Elbow	-0.0088	0.019816
216	Right Elbow	-0.0136	7.66E-07
217	Left Forearm	-0.0115	0.001026
218	Lower Back	-0.01389	9.91E-16
219	Lower Back	-0.01229	2.36E-12
220	Right Forearm	-0.01599	3.36E-08
221	Left Wrist	-0.01602	1.57E-10
222	Left Hip	-0.0107	1.37E-06
223	Buttocks	-0.01346	2.49E-11
224	Buttocks	-0.009	6.44E-05
225	Right Hip	-0.00768	0.002969
226	Right Wrist	-0.01833	8.48E-15
227	Left Hand	-0.02004	9.40E-19
228	Left Upper Leg	-0.01085	3.13E-05
229	Right Upper Leg	-0.00512	1
230	Right Hand	-0.02125	1.44E-22
231	Left Knee	-0.00966	2.27E-04
232	Right Knee	-0.00962	2.22E-04
233	Left Lower Leg	-0.0077	0.029588
234	Right Lower Leg	-0.00714	0.067027
235	Left Ankle	-0.00804	0.010443
236	Right Ankle	-0.00853	0.0026
237	Left Foot	-0.01516	2.75E-12
238	Right Foot	-0.01503	1.94E-12

### Co-occurrence of CBM location endorsement

**Fig 7:**
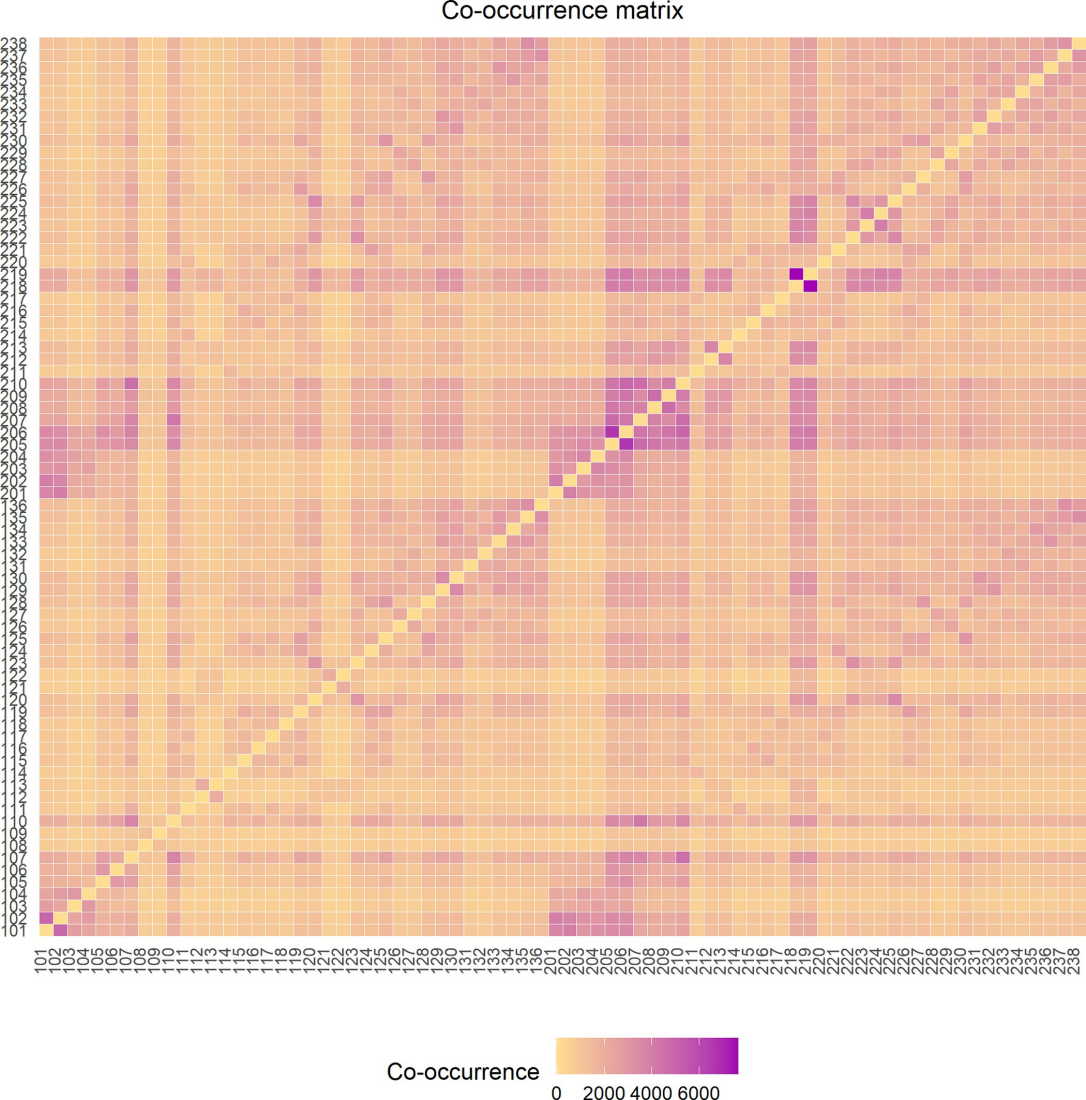
A co-occurrence heatmap illustrating the number of times each CBM area is endorsed concurrently with every other area. Cells are colored according to the number of times that any two locations were endorsed together by a patient, with lighter hues indicating more common endorsement in the dataset.

To assess co-occurrence, the comp_cooccurrence() function was used to generate a matrix of all possible combinations of the 74 CBM locations and the number of times that any two locations were endorsed together by a patient. The plot_cooccurrence() function was then used to visualize the cooccurrence matrix as a heatmap (**Fig 7**). The three most co-endorsed pairs of locations (as shown in **Table 4**) were: 218 with 219 which comprise the lower back, 205 with 206 or the back of the neck, and 101 with 102 which corresponds to the top of the head. These results are consistent with prior clinical work [32,33,36–39].

**Table 4:** The ten most co-endorsed locations of the CBM (of 5,402 possible) using data collected during the validation study.

Location Code 1	Location Code 2	Anatomical Description	Co-occurrence
218	219	Lower Back, Lower Back	7638
205	206	Neck, Neck	6637
101	102	Top of the Head, Top of the Head	4926
206	210	Neck, Right Shoulder	4925
205	207	Neck, Left Shoulder	4753
207	210	Left Shoulder, Right Shoulder	4701
208	209	Upper Back, Upper Back	4675
107	210	Right Shoulder (Front and Back)	4576
206	209	Neck, Upper Back	4394
205	210	Neck, Right Shoulder	4342

### Availability and future directions

The open-source CHOIRBM software package (implemented in R) available for download via CRAN, and the development version is available on Github (http://github.com/emcramer/CHOIRBM). Additionally, installation instructions, tutorials, and detailed vignettes are available at https://cran.r-project.org/web/packages/CHOIRBM/. The ggplo2 R package, used with CHOIRBM for plotting, is available via CRAN (https://cran.r-project.org/web/packages/ggplot2/index.html) and Github (https://github.com/tidyverse/ggplot2).

The CHOIRBM package contains a collection of statistical and plotting functions for visualizing body map data collected with the Collaborative Health Outcomes Information Registry’s Body Map (CBM). The R functions include tools for data formatting and pre-processing, statistical analysis, and comparisons between CBMs of different groups, co-occurrence analysis of pain locations, and visualization of the CBM. There are several extensions of the CHOIRBM package which may naturally follow, such as developing and deploying a user interface (e.g., a Shiny application) for researchers, adding statistical tests and methods such as ANOVA, textual annotations for each CBM location, or building direct connectivity and data import for web-based institution-specific electronic data capture systems (beyond CHOIR and REDCap). The grammar of graphics approach to CHOIRBM’s implementation means user’s may easily customize output for specific applications, and the open-source distribution will allow researchers to contribute their extensions to the public code repository. Finally, suggestions for new functionality may be made through the ‘Issues’ tab of the CHOIRBM GitHub repository (http://github.com/emcramer/CHOIRBM).
